# High intensity smoking cessation interventions: Cardiac patients of low socioeconomic status and low intention to quit profit most

**DOI:** 10.1007/s12471-016-0906-7

**Published:** 2016-10-17

**Authors:** N. Berndt, H. de Vries, L. Lechner, F. Van Acker, E. S. Froelicher, F. Verheugt, A. Mudde, C. Bolman

**Affiliations:** 1 Department of Psychology and Educational Sciences, Open University of the Netherlands, Heerlen, the Netherlands; 2 Cellule d’Expertise Médicale, General Inspectorate of Social Security, Ministry of Social Security, Luxembourg, Luxembourg; 3 Department of Health Promotion, Caphri School for Public Health and Primary Care, Maastricht University, Maastricht, the Netherlands; 4 Department of Remedial Education, Artesis Plantijn Hogeschool Antwerpen, Antwerpen, Belgium; 5 Department of Physiological Nursing, School of Nursing, and Department of Epidemiology and Biostatistics, School of Medicine, University of California San Francisco, California, USA; 6 Department of Cardiology, Onze Lieve Vrouwe Gasthuis, Amsterdam, the Netherlands

**Keywords:** Coronary heart disease, Smoking cessation, Telephone counselling, Face-to-face counselling, Socioeconomic status, Intention to quit

## Abstract

**Background:**

Without assistance, smokers being admitted to the hospital for coronary heart disease often return to regular smoking within a year.

**Objective:**

This study assessed the 12-month effectiveness of a telephone and a face-to-face counselling intervention on smoking abstinence among cardiac patients. Differential effects for subgroups varying in their socioeconomic status and intention to quit smoking were also studied.

**Methods:**

A randomised controlled trial was used. During hospital stay, smokers hospitalised for coronary heart disease were assigned to usual care (*n* = 245), telephone counselling (*n* = 223) or face-to-face counselling (*n* = 157). Eligible patients were allocated to an intervention counselling group and received nicotine patches. After 12 months, self-reported continued abstinence was assessed and biochemically verified in quitters. Effects on smoking abstinence were tested using multilevel logistic regression analyses applying the intention-to-treat approach.

**Results:**

Compared with usual care, differential effects of telephone and face-to-face counselling on continued abstinence were found in patients with a low socioeconomic status and in patients with a low quit intention. For these patients, telephone counselling increased the likelihood of abstinence threefold (OR = 3.10, 95 % CI 1.32–7.31, *p* = 0.01), whereas face-to-face counselling increased this likelihood fivefold (OR = 5.30, 95 % CI 2.13–13.17, *p* < 0.001). Considering the total sample, the interventions did not result in stronger effects than usual care.

**Conclusion:**

Post-discharge telephone and face-to-face counselling interventions increased smoking abstinence rates at 12 months compared with usual care among cardiac patients of low socioeconomic status and low quit intentions. The present study indicates that patients of high socioeconomic status and high quit motivation require different cessation approaches.

## Background

Although studies have estimated up to 40 % reduced risks of recurrent coronary events and subsequent mortality in patients who quit smoking after a coronary event [1], one out of two patients still persist in smoking or relapse after hospital discharge [2–4]. Behavioural interventions initiated during hospital admission with regular follow-up contacts for at least one month after discharge yielded significantly higher long-term abstinence rates than brief interventions in hospitalised patients [5]. Studies testing such interventions in cardiac patients also demonstrated high rates of sustained abstinence [6–10]. Combining intensive behavioural counselling with pharmacotherapy further increases effectiveness [5, 11, 12]. Despite accumulating evidence of success of such interventions, no studies have compared different counselling modalities for determining which intervention is most successful for whom. Reviews showed that telephone and face-to-face counselling are similarly effective for smokers in general, though telephone counselling is generally less intensive than face-to-face counselling [13, 14]. Yet, these delivery methods have not yet been experimentally tested against each other. Identifying which intervention is most effective for which patient subgroup can provide valuable information for their implementation.

Relevant in this context are subgroups of patients that are at higher risk of continued smoking, i. e. those with a low socioeconomic status (SES). Socioeconomic inequalities in health behaviour persist in coronary heart disease [15]. Studies have shown that individuals with a lower SES have a less favourable profile towards smoking than high SES patients due to a lower self-efficacy towards non-smoking, a less favourable social environment, less social support, and more stress and negative affect leading to fewer successful quit attempts [16]. Moreover, smokers with a low SES have been found to profit least from cessation methods due to, among other reasons, a lack of motivation to quit [15, 17–19]. Studies therefore suggest the need for relatively intensive interventions for these groups [20]. In contrast, smokers with a high SES are recognised as more successful in quitting due to a higher intention to quit and may therefore profit sufficiently from somewhat less intensive interventions [17].

The present study compared two different smoking cessation counselling interventions initiated during hospital admission with usual care among subgroups of cardiac patients in a multicentre randomised controlled trial. It was hypothesised that face-to-face counselling increases the proportion of patients who abstain from smoking among low SES patients and patients with a low motivation to quit, whereas telephone counselling would be sufficient to reduce smoking in patients with a high SES and a high motivation to quit 12 months after hospital discharge.

## Methods

### Setting and design

A randomised controlled trial with sequential cross-over randomisation at the cardiac ward level of eight hospitals throughout the Netherlands was used. The Medical Research Ethics Committee of the VU Medical Centre Amsterdam and the review board of each hospital approved the study protocol (Trial Registration: NTR2144).

### Study population

**Fig. 1:**
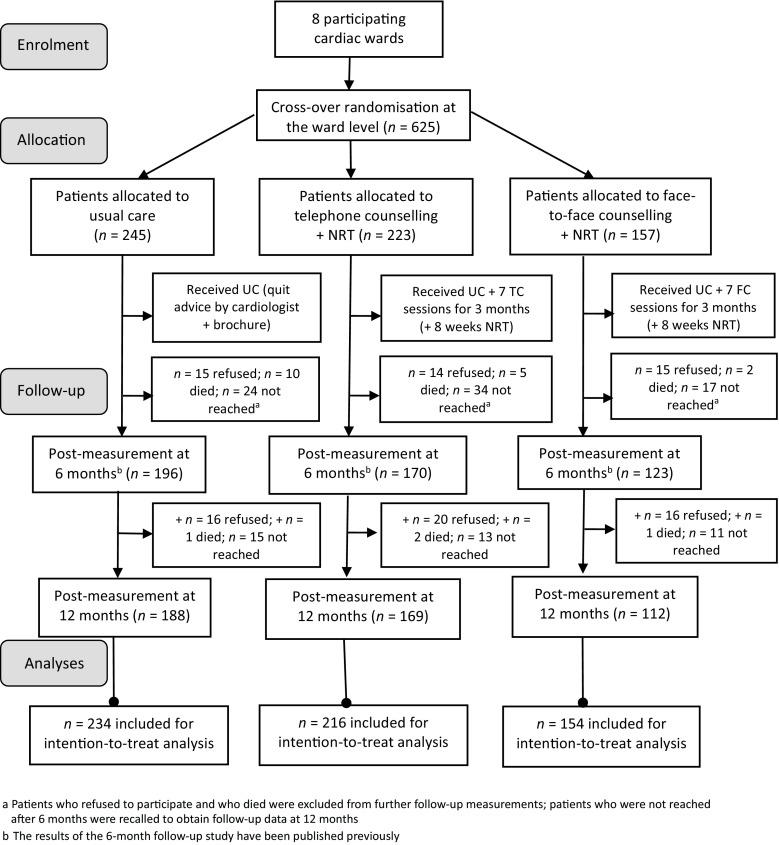
Flow diagram of the experimental study evaluating two smoking cessation counselling interventions in cardiac patients. *UC* usual care, *NRT* nicotine replacement therapy, in this trial nicotine patches only given to patients who agreed to use them and without any contraindications indicated by the cardiologist, *TC* telephone counselling, *FC* face-to-face counselling

From December 2009 through June 2011, ward nurses recruited eligible patients at the bedside. Eligible patients were ≥18 years of age, smoked ≥5 cigarettes per day or had quit smoking less than 4 weeks prior to admission, and had been admitted to the cardiac ward for less than 4 days for an acute coronary syndrome, stable angina, or other forms of heart disease following the International Classification of Diseases-10 [21]. Exclusion criteria were being unable to speak and/or read Dutch, not owning a telephone, a medically unstable situation, and cognitive impairment. Patients provided written informed consent. Fig. 1 presents inclusion rates.

### Interventions

#### Usual care

Usual care consisted of an assessment of the smoking behaviour, provision of advice to quit by cardiologists or ward nurses, and delivery of a general smoking cessation brochure.

#### Protocol-based interventions: telephone and face-to-face counselling

**Fig. 2:**
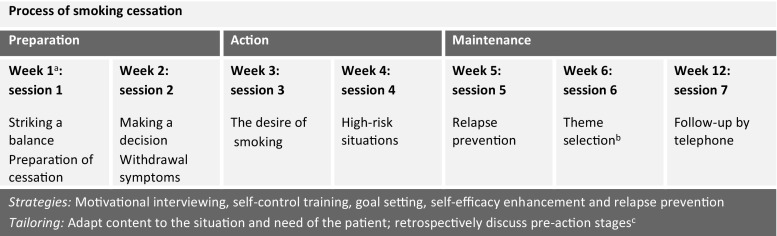
Intervention protocols of telephone counselling and face-to-face counselling. ^a^Each telephone counselling session was designed to last 15 min and each face-to-face session 30–45 min besides the last follow-up by telephone (also 15 min). ^b^For telephone counselling, the themes of sessions 5 and 6 were discussed the other way round. ^c^Many patients are already in the action stage because they quit at hospital admission

Details of the intervention protocols have been reported elsewhere [22]. The telephone and face-to-face counselling intervention had an inpatient and outpatient phase. The inpatient phase incorporated the Ask-Advise-Refer strategy [23, 24] in which patients’ smoking behaviour was assessed, smokers were advised to quit, and referred to outpatients’ smoking cessation counselling, either by telephone or face-to-face. Interventions started within one week of patient enrolment and had a comparable structure and content. The counsellors tailored the counselling to the patient’s stage in the quitting process (Fig. 2). Telephone counselling was provided by counsellors from the Dutch Expert Centre for Tobacco Control and lasted 15 min per call. Face-to-face counselling was provided by cardiac nurses who, for the purpose of the study, were qualified as smoking cessation counsellors. Face-to-face counselling lasted 30–45 min per session. In each session, patients were counselled by the same counsellor. The counsellors worked with a protocol and following each session, they completed a registration form (one per patient) on which the date, the duration, and important remarks on the session were noted. Upon admission, ward nurses also provided nicotine patches for eight weeks at no cost to patients eligible for their use, as indicated by the cardiologist.

### Data collection at baseline

At baseline nurses recorded patient data and administered a questionnaire to patients for assessing various socio-demographics, diagnosis and other disease-related factors, psychosocial factors, and smoking-related factors such as current smoking behaviour, past smoking habits, and nicotine dependence level [25, 26]. The psychosocial factors assessed were levels of anxiety (α = 0.79) and depression (α = 0.81) with the Hospital Anxiety and Depression Scale (HADS) [27], self-efficacy towards smoking abstinence in various situations (α = 0.93) [28], and intention to quit (α = 0.86) [29]. For assessing intention to quit, patients had to indicate how strongly they intended to quit smoking and how likely it was that they would refrain from smoking after hospital discharge with answers ranging from ‘not likely’ (1) to ‘very likely’ (5) for both items [29].

### Data collection at follow-up

#### Outcome measurement

Outcomes after 12 months were obtained in structured telephone interviews by an independent call agency, for which patients received a reminder by post. The primary outcome measure was continued abstinence according to the intention-to-treat scenario. To determine the duration of smoking abstinence in those patients who reported to have quit, the quit date indicated by patients was subtracted from the date of the telephone interview, and this period had to be at least 240 days (accounting for the 3‑month intervention duration and some variation) to be classified as being continuously abstinent from smoking.

#### Biochemical validation

Patients who indicated not smoking at 12-month follow-up (*n* = 187) were invited to the hospital for biochemical validation using the NicAlert® test strips for assessing cotinine in saliva specimens. Tests were conducted by nurses in 79 patients. Eight patients completed the test at home because they were not able to attend the hospital. The other patients (*n* = 100) did not complete the test because they did not show up in the hospital or refused to do the test. A cut-off point of ≥2 for saliva indicated tobacco smoking [30]. In 90 % (*n* = 79) of all the tests the result of the NicAlert® test was ≤2 and in agreement with the self-reports. For the 10 % (*n* = 8) in which cotinine was detected, patients were treated as smokers.

### Statistical analyses

Baseline characteristics of the three groups were compared using ANOVAs and χ^2^ tests. An attrition analysis was conducted using a logistic regression analysis to determine possible selective dropout at follow-up excluding patients who died (*n* = 21). The χ^2^ tests subsequently assessed continued abstinence rates for each group at 12-month follow-up.

The main analysis concerned a generalised linear mixed model (GLMM) to account for correlated data structure and possible hospital-level variation. The GLMM tested the effects of the interventions and corrected for possible confounding factors. Differential effects for SES and intention to quit were also tested. Therefore, SES was dichotomised in low versus high SES on the basis of its median score whereas intention to quit was transformed into z‑scores. To obtain the most parsimonious model, a hierarchical top-down elimination procedure was used removing non-significant covariates (*p* < 0.05) until only variables remained that made significant contributions to the prediction of continued abstinence, plus baseline differences. When differential effects were detected (*p* ≤ 0.10), GLMM analyses were repeated for subgroups changing the reference category in categorical variables, or changing the value of the moderator to low (z-score −1), moderate (z-score), and high (z-score +1) in continuous variables. This was done to test the simple effects of the interventions for the respective subgroup. Post-hoc χ^2^ tests were conducted to assess the difference in effect for the moderator analysing continued abstinence rates for each group. Data were analysed using the ‘intention-to-treat’ approach.

## Results

As summarised in Table 1, the usual care group had significantly lower nicotine dependence than the face-to-face counselling group, and there was a significantly higher rate of patients in the usual care group who had made one or more attempts to quit over the past 12 months compared with the telephone counselling group.

**Table 1:** Baseline characteristics of patients enrolled in the study (*n* = 625)^a^

	UC (*n* = 245)	TC (*n* = 223)	FC (*n* = 157)	χ²/F ratio	*p*
**Sociodemographic factors**					
Age; M (SD)	56.08 (10.96)	55.31 (10.53)	56.54 (10.57)	0.65	0.521
Sex (male); *n* (%)	183 (74.7)	163 (73.1)	111 (70.7)	0.77	0.678
*Marital status; n (%)*					
Married with/without children	161 (67.6)	151 (68.9)	102 (66.2)	0.31	0.857
Single/divorced/widow	76 (32.1)	68 (31.1)	52 (33.8)	–	–
*Education level* ^b^ *; n (%)*					
Low education	99 (41.8)	84 (38.2)	64 (41.6)	2.37	0.667
Intermediate education	85 (35.9)	88 (40.0)	63 (40.9)	–	–
High education	53 (22.4)	48 (21.8)	27 (17.5)	–	–
**Clinical factors**					
*Disease diagnosis; n (%)*					
ACS^c^	212 (86.9)	192 (86.1)	131 (83.4)	5.18	0.270
Stable angina	16 (6.6)	23 (10.3)	14 (8.9)	–	–
Other/unspecified diagnosis	16 (6.6)	8 (3.6)	12 (7.6)	–	–
Previous hospital admission; *n* (%)	49 (20.4)	37 (16.7)	38 (24.5)	3.45	0.179
*Blood pressure; M (SD)*					
Systolic	123.51 (19.07)	123.89 (17.88)	124.07 (16.38)	0.05	0.950
Diastolic	71.61 (11.88)	72.60 (10.86)	72.92 (10.69)	0.76	0.468
Total/HDL cholesterol ratio; M (SD)	4.80 (1.93)	4.66 (1.58)	4.73 (1.57)	0.38	0.686
Body mass index; M (SD)	26.52 (4.10)	27.09 (5.69)	26.87 (4.81)	0.76^†^	0.470
Cardiac rehabilitation; *n* (%)	105 (44.9)	99 (45.8)	58 (37.7)	8.54	0.074
**Smoking-related factors**					
Nicotine dependence^d^; M (SD)	5.09 (2.34)	5.31 (2.10)	5.69 (2.00)	3.56	0.029*
Average cigarettes per day; M (SD)	19.75 (10.38)	21.13 (13.79)	22.28 (11.67)	2.17	0.116
7-day abstinence at admission; *n* (%)	89 (36.8)	66 (29.9)	46 (29.7)	3.28	0.194
Previous attempt(s) to quit; *n* (%)	87 (36.6)	56 (25.2)	46 (29.7)	7.03	0.030*
Smoking partner; *n* (%)	100 (41.3)	81 (36.5)	54 (34.8)	2.21	0.697
**Psychosocial factors**					
HADS-Anxiety^e^; M (SD)	6.10 (4.06)	6.70 (4.18)	6.84 (4.14)	1.92	0.148
HADS-Depression^f^; M (SD)	5.56 (4.09)	5.22 (4.10)	5.51 (4.10)	0.45	0.640
Self-efficacy^g^; M (SD)	2.69 (0.76)	2.75 (0.72)	2.57 (0.78)	2.24^†^	0.108
Intention to quit^h^; M (SD)	7.53 (2.29)	7.50 (2.13)	7.48 (2.02)	0.02	0.980

*UC* usual care, *TC* telephone counselling, *FC* face-to-face counselling

^a^Missing data are excluded (pairwise deletion) so *n* < 625 for some analyses

^b^Low education = primary and basic vocational schools; intermediate education = secondary vocational schools and high school degrees; high education = higher vocational school degrees, college or university degrees

^c^ACS = acute coronary syndrome, includes unstable angina pectoris and (non) ST elevation myocardial infarction

^d^Range from 0 = low nicotine dependence to 10 = high nicotine dependence

^e^Range from 0 = low anxiety level to 21 = high anxiety level

^f^Range from 0 = low depression level to 21 = high depression level

^g^Range from 0 = low self-efficacy to 4 = high self-efficacy towards smoking abstinence

^h^Range from 2 = weak intention to 10 = strong intention to quit smoking

† For non-equal variances between the groups, the Brown-Forsythe statistic and *p *value are reported

*p < 0.05 (significantly different to referent group (usual care))

Post-hoc tests: For nicotine dependence, Tukey post-hoc tests reveal that FC differs significantly from UC. For attempts to quit over the past 12 months, χ² analysis reveals that TC and UC differ significantly from each other

### Differential effects for telephone and face-to-face counselling on smoking abstinence at 12-month follow-up

No difference in attrition rates was observed between the three groups at follow-up (Fig. 1). However, there were trends that patients were more likely to be lost to follow-up when they had previously been admitted to the hospital (*OR* = 1.62, 95 % CI 1.00–2.61, *p* = 0.05) and when they had made one or more attempts to quit in the past (*OR = *1.51, 95 % CI 0.98–2.33, *p* = 0.06).

Crude quitting rates revealed that 26.9 % of the patients in the usual care group continued to be abstinent compared with 34.7 % in the telephone counselling and 33.1 % in the face-to-face counselling group at 12-month follow-up, respectively (χ²(2) = 3.50, *p* = 0.17). As depicted in Table 2, the GLMM analysis revealed borderline significant differential effects of the interventions on continued abstinence by SES (*p* = 0.09) and by intention to quit (*p* = 0.08) (first model). After removing non-significant covariates the final GLMM model yielded significant positive differential effects for both telephone and face-to-face counselling compared with usual care on continued abstinence for patients with a low SES and patients with a low intention to quit, the effect being largest for face-to-face counselling (telephone counselling: *OR* = 3.10, 95 % CI 1.32–7.31, *p* = 0.01; face-to-face counselling: *OR* = 5.30, 95 % CI 2.13–13.17, *p* < 0.001). For the interventions compared with usual care, repeating the analysis to assess the nature of the differential effects (not shown in table) yielded significant odds ratios on continued abstinence in case of a low SES and a moderate intention to quit (telephone counselling: *OR* = 2.04, 95 % CI 1.10–3.81, *p* = 0.03; face-to-face counselling: *OR* = 2.57, 95 % CI 1.32–5.01, *p* = 0.01). No differential effects of telephone and face-to-face counselling compared with usual care on continued abstinence were found for high SES patients and patients with a high intention to quit (telephone counselling: *OR* = 0.82, 95 % CI 0.39–1.72, *p* = 0.60; face-to-face counselling: *OR* = 0.46, 95 % CI 0.20–1.09, *p* = 0.08). Replicating the analysis for other scenarios (low SES, high quit intention; high SES, low quit intention; high SES, moderate quit intention) yielded no differential intervention effect either. Nonetheless, to compare the effects of telephone versus face-to-face counselling, further GLMM analyses were performed using telephone counselling as the reference group. With a high SES and a high intention to quit, results revealed no significant differential effects of face-to-face counselling (*OR = *0.56, 95 % CI 0.24–1.34; *p* = 0.20) or usual care (*OR* = 1.22, 95 % CI 0.58–2.56, *p* = 0.60) compared with telephone counselling. Repeating the analysis for low SES patients and for patients with a low intention to quit revealed that usual care significantly decreased the likelihood of continued abstinence compared with telephone counselling (*OR* = 0.32, 95 % CI 0.14–0.76, *p* = 0.01). Face-to-face counselling for these patients did not have a significant differential effect when compared with telephone counselling (*OR* = 1.71, 95 % CI 0.77–3.80; *p* = 0.19). All final GLMMs revealed that age, having received cardiac rehabilitation, and not having made any previous attempts to quit smoking significantly increased the likelihood of continued abstinence, whereas a higher nicotine dependence decreased its likelihood.

**Table 2:** Differential effects of the telephone and face-to-face counselling intervention on continued abstinence from smoking for patients with a low SES and patients with a low quit intention at 12-month follow-up (intention-to-treat) (*n* = 604)^a^

	First model (*n* = 568)			Final model (*n* = 579)		
Variables	OR	95 % CI	*p*	OR	95 % CI	*p*
Telephone counselling	3.07	[0.97,9.73]	0.057	3.10	[1.32,7.31]	0.010
Face-to-face counselling	5.61	[1.85,17.04]	0.002	5.30	[2.13,13.17]	0.000
Age	1.02	[1.02,1.04]	0.029	1.03	[1.01,1.05]	0.009
Sex (male)	0.98	[0.63,1.54]	0.994	–	–	–
Marital status (married)	1.19	[0.75,1.87]	0.462	–	–	–
SES (high education) ^b^	1.87	[0.94,3.72]	0.075	1.87	[0.97,3.62]	0.064
*Disease diagnosis*						
ACS^c^	1.36	[0.50,3.67]	0.543	–	–	–
Unstable angina	2.23	[0.68,7.31]	0.184	–	–	–
Cardiac rehabilitation	2.34	[1.56,3.52]	0.000	2.55	[1.73,3.75]	0.000
Previous admission	1.35	[0.79,2.30]	0.280	–	–	–
Nicotine dependence	0.92	[0.84,1.01]	0.081	0.91	[0.83,0.99]	0.026
7-day abstinence at admission	1.36	[0.88,2.08]	0.163	–	–	–
Previous attempt(s) to quit	1.54	[0.99,2.40]	0.056	1.68	[1.10,2.57]	0.018
Smoking partner	0.72	[0.47,1.10]	0.128	–	–	–
HADS-Anxiety	1.02	[0.96,1.09]	0.515	–	–	–
HADS-Depression	0.94	[0.88,1.01]	0.091	–	–	–
Self-efficacy	1.25	[0.93,1.68]	0.148	–	–	–
Intention to quit	1.29	[1.07,1.56]	0.009	1.43	[1.20,1.71]	0.000
Condition x SES^d^	–	F = 2.36	0.094	–	F = 1.86	0.157
TC x high SES	0.54	[0.21,1.38]	0.195	0.61	[0.25,1.51]	0.284
FC x high SES	0.33	[0.12,0.91]	0.032	0.37	[0.14,0.99]	0.048
Condition x intention to quit^e^	–	F = 2.47	0.083	–	F = 3.32	0.037
TC x high intention to quit	0.73	[0.43,1.23]	0.234	0.66	[0.40,1.10]	0.111
FC x high intention to quit	0.53	[0.30,0.93]	0.027	0.49	[0.28,0.84]	0.010
	–	ICC = 0.004	0.408	–	ICC = 0.008	0.413

*SES* socioeconomic status; *TC* telephone counselling; *FC* face-to-face counselling; *ICC* intraclass correlation coefficient

Five dummy variables coding time effects were entered simultaneously with all other variables, but their coefficients are not reported here (1 = Dec 2009–Jan 2010; 2 = Feb–June 2010; 3 = July–Nov 2010; 4 = Dec 2010–Jan 2011; 5 = Feb–June 2011). The Model uses reference groups for categorical variables [condition = usual care; time effects = Feb–June 2011;﻿ sex = female gender; SES = high education; diagnosis = non-specified diagnosis; cardiac rehabilitation = no; previous admission = yes; 7‑day abstinence at admission = no; previous quit attempt = no; smoking partner = no]

^a^Sample including patients lost at follow-up as smokers. Patients with missing data on predictor variables are excluded (listwise deletion) so *n* < 604 for the analyses; *n* = 21 died and were excluded

^b^Socioeconomic status (SES) was derived from education and categorised as primary, intermediate or tertiary education

^c^ACS = acute coronary syndrome, includes unstable angina pectoris and (non) ST elevation myocardial infarction

^d^Only two-way interactions were tested, thus the differential effects of SES and intention need to be treated separately. Combinations yield similar results when three-way interactions are present

^e^For the interactions with intention to quit, the continuous scale scores were transformed into z‑scores

### Smoking abstinence rates for subgroups at 12-month follow-up

Post-hoc χ^2^ tests (Table 3) analysing continued abstinence rates for low versus high SES patients showed significantly higher rates of quitters in the telephone and face-to-face counselling group compared with the usual care group for low SES patients. This was not found for high SES patients. Repeating the analysis for patients with a low intention to quit revealed significantly more quitters in the telephone counselling and face-to-face counselling group compared with the usual care group, but not among patients with a high quit intention.

**Table 3:** Continued abstinence rates for each group specified by SES and intention to quit at 12-month follow-up (intention-to-treat)

**Continued abstinence rates for low and high SES patients (** ***n*** ** = 591)** ^**a**^							
*Low SES*	*% CA*	*χ²*	*p*	*High SES*	*% CA*	*χ²*	*p*
UC (*n* = 130)	20.0 (*n* = 26)^b^	–	–	UC (*n* = 95)	37.9 (*n* = 36)^b^	–	–
TC (*n* = 130)	33.8 (*n* = 44)^c^	8.45	0.015	TC (*n* = 84)	36.9 (*n* = 31)^b^	1.44	0.486
FC (*n* = 90)	35.6 (*n* = 32)^c^	–	–	FC (*n* = 62)	29.0 (*n* = 18)^b^	–	–
**Continued abstinence rates for patients with low and high quit intentions (** ***n*** ** = 598)** ^**a**^							
*Low intention to quit*	*% CA*	*χ²*	*p*	*High intention to quit* ^*d*^	*% CA*	*χ²*	*p*
UC (*n* = 95)	13.7 (*n* = 13)^b^	–	–	UC (*n* = 137)	36.5 (*n* = 50)^b^	–	–
TC (*n* = 92)	27.2 (*n* = 25)^c^	7.91	0.019	TC (*n* = 121)	40.5 (*n* = 49)^b^	0.93	0.627
FC (*n* = 68)	30.9 (*n* = 21)^c^	–	–	FC (*n* = 85)	34.1 (*n* = 29)^b^	–	–

*SES* socioeconomic status*; CA* continued abstinence; *UC* usual care; *TC* telephone counselling; *FC* face-to-face counselling

^a^Missing data are excluded so *n* < 604 for some analyses; *n* = 21 died and were excluded

^b,c^Pairwise analyses for each scenario: Groups with the same superscript do not differ from each other at *p* < 0.05, other groups do differ

^d^For the purpose of the comparisons, intention to quit was dichotomised on the basis of its median score

## Discussion

Despite efforts in optimising smoking cessation programs, the overall percentage of continued smoking abstinence is still disappointing in the Netherlands [4] and therefore remains an ongoing challenge. This study showed that intensive counselling interventions initiated upon admission and continued for a prolonged period post-discharge result in substantially higher long-term smoking abstinence rates in cardiac patients at high risk of continued smoking, i. e. with a low SES and with a low to moderate intention to quit. The short-term effectiveness study after six months revealed that the interventions were both overall effective considering continued abstinence rates, and significant conditional effects of the interventions were found in patients with a lower SES [31]. Although the intervention effects diminished after 12 months for the total sample, among low SES patients and patients with a low intention to quit participation in telephone counselling increased the likelihood of smoking abstinence threefold, whereas participation in face-to-face counselling increased this likelihood fivefold when compared with usual care. These results are promising since previous studies showed that this particular patient group often persists in smoking [15–20].

The effectiveness of our study is likely to evolve from the Ask-Advise-Refer strategy, the one week post-discharge start of the counselling, the use of motivational interviewing strategies, and the counselling provision by trained smoking cessation professionals outside the cardiac ward. Low SES patients and patients with a lower intention to quit may have especially profited from face-to-face counselling because these counselling sessions had a longer duration than the telephone sessions. One possible explanation for the absence of effects among patients with a more favourable profile might be that high SES patients already had high intentions and generally did well in their process of quitting smoking, thus there was limited added value of participating in telephone or face-to-face counselling. Moreover, it could be that those patients had quit immediately after their event without support, perhaps not requiring any intervention to remain successfully abstinent, as recently shown by Snaterse et al. [4]. As indicated by the earlier studies in general populations of smokers [20], low SES groups profited most from high intensity face-to-face interventions.

Misrepresentation of smoking status based on self-report was found to be 10 % which is relatively low [32]. However, the biochemical validation test was only realised in 44 % of all self-reported quitters which is most likely due to practical limitations in performance of the test [33]. Since it is likely that self-reported outcomes were not completely accurate in patients who did not undergo validation, non-smoking status may have been overestimated. We corrected for this by using the intention-to-treat approach which may have yielded an underestimation of the intervention effects compared with earlier studies [7, 8]. Another source for debate is the cut-off level that we used to discriminate smokers from non-smokers. As suggested previously [30, 33], a higher cut-off than proposed by the manufacturer was chosen due to a present risk of inaccuracy of the NicAlert® saliva test result, the potential use of nicotine replacement therapies, and passive exposure to tobacco smoke in the days before the test to be able to discriminate smokers from non-smokers more clearly.

Other study limitations became apparent. It is conceivable that selection bias occurred due to baseline differences between the three groups and due to patients who refused to participate in the study. Accurate insight cannot be provided into this matter because data of eligible patients who refused participation were not registered. Moreover, as recent quitters participated in the study, their quitting cannot be attributed solely to the interventions. However, since this applied equally to the control group and the intervention groups, it was still possible to assess the additional effects of the interventions. It can also be presumed that the telephone counsellors and ward nurses trained as face-to-face counsellors differed in terms of previous experience in delivering smoking cessation counselling. Since there is evidence that interventions delivered by multiple health care professionals of different backgrounds are susceptible to greater variability [34], confounding might have been introduced that could have contributed to the differences in intervention effects.

Our study supports the long-term effectiveness of intensive smoking cessation counselling interventions for cardiac patients with a less favourable profile. These interventions are particularly effective in low SES patients and patients with weak intentions to quit smoking. Face-to-face counselling revealed the greatest effects in these patients. We found no evidence that smoking cessation counselling provided face-to-face or by telephone is beneficial for patients with a high SES and a high intention to quit. Although these types of intensive interventions may reduce socioeconomic disparities in smoking, we conclude that different cessation approaches are required for patients differing in their SES and their motivation to quit. Future studies need to investigate which intervention approach is particularly suitable for high SES patients with high intentions to quit to improve their abstinence rates.
